# Recurrent Transient Ischemic Attacks While on Hemodialysis: A Case Report

**DOI:** 10.7759/cureus.83002

**Published:** 2025-04-25

**Authors:** Pallavi Shirsat, Kunal Sonavane, Bhawna Agarwal, Gautam Agrawal

**Affiliations:** 1 Nephrology, Minden Medical Center, Minden, USA; 2 Internal Medicine, Willis Knighton Health, Bossier City, USA; 3 Internal Medicine, University of Pittsburgh Medical Center McKeesport Hospital, McKeesport, USA; 4 Nephrology, Independence Health System, Greensburg, USA

**Keywords:** carotid artery stenosis, cerebral blood flow shunting, dialysis challenges, dialysis disequilibrium, end stage renal disease (esrd), hemodialysis related, recurrent tia, recurrent transient ischemic attacks, transient ischemic attacks

## Abstract

Cerebrovascular accidents (CVAs) remain a crucial cause of morbidity and mortality while posing a significant burden to public health systems worldwide. Its incidence is higher in elderly patients with end-stage renal disease (ESRD) on hemodialysis due to concomitant conditions (advanced age, diabetes, cardiovascular disease, hypertension), altered cerebral blood flow, and frequent use of anticoagulants during hemodialysis treatments.

In this report, we discuss a case of an 85-year-old female patient with ESRD on hemodialysis who started having recurrent episodes of transient ischemic attacks (TIA) that coincided with her dialysis treatments. Cerebral blood flow shunting during dialysis sessions was thought to be the likely cause of these events. After multiple failed attempts to convince the patient to transition to peritoneal dialysis, her dialysis prescription was modified to shorter duration treatments. With this modification, she experienced no further TIA episodes. This highlights the importance of an individualized approach to management decisions when there is a lack of clear treatment guidelines.

## Introduction

Cerebrovascular accidents (CVAs) are one of the leading causes of morbidity and mortality worldwide and pose an increased risk of long-term disability, contributing to high public health costs [1]. Elderly patients with end-stage renal disease (ESRD) on hemodialysis are inherently at high risk for such unfortunate occurrences due to a multitude of factors like advanced age, comorbidities that are frequent in the dialysis population, for instance, hypertension, diabetes, cardiovascular disease, atrial fibrillation, obesity, physical inactivity, shunting of cerebral blood flow during hemodialysis, and use of anticoagulants during dialysis treatments [2].

This report presents a case involving a hemodialysis patient who had recurrent episodes of transient ischemic attacks (TIAs) that happened exclusively during dialysis. After a series of tests, it was determined that these episodes were occurring due to carotid stenosis. Cerebral blood flow shunting that can happen when patients are on hemodialysis can heighten the risk of acute cerebral ischemia. Managing these episodes while continuing life-prolonging treatment of dialysis can be particularly difficult in the absence of established guidelines. In this case, we successfully prevented further TIA episodes through adjustments of the dialysis prescription.

## Case presentation

The patient was an 85-year-old woman with hypertension, type 2 diabetes leading to diabetic kidney disease, and ESRD on hemodialysis for the last 10 months. She reported no history of cardiac arrhythmias. During one of her dialysis treatments at an outpatient dialysis unit, she had a sudden onset of loss of consciousness, where she was noted to have facial droop to the right. This was associated with tonic posturing that lasted for about two to three minutes and was followed by slurred speech. Her heart rate and blood pressure remained stable during this episode. The patient’s blood glucose during this event was within normal limits. Her dialysis treatment was terminated at that point. A few minutes after the termination of dialysis, she regained consciousness, and her neurologic deficit resolved. She did not have loss of bowel or bladder control, nor did she have tongue bite. Differential diagnoses of TIA versus seizures were considered. She was subsequently sent to the emergency room.

**Figure 1:**
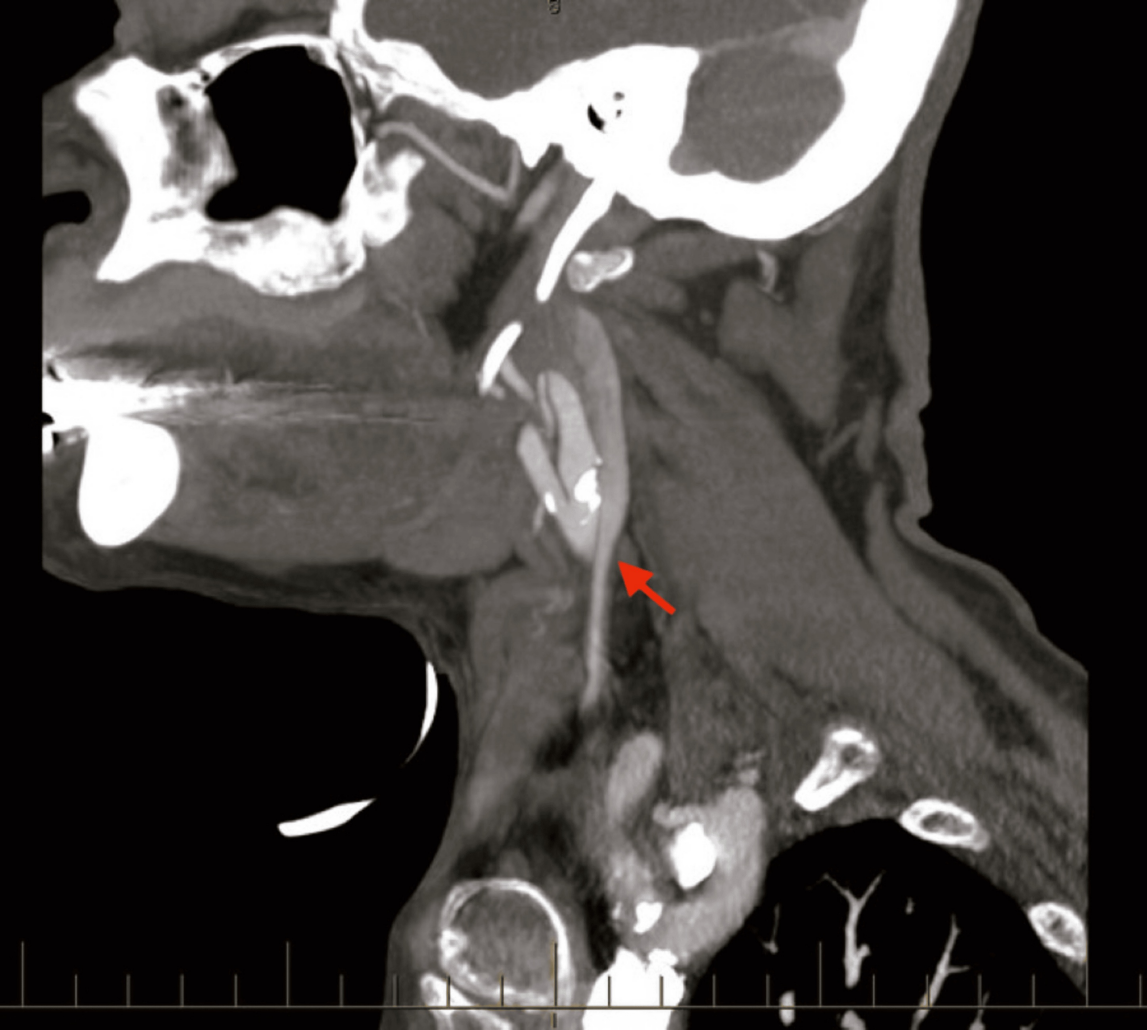
Sagittal section of CT angiography of the neck showing 70% stenosis of the left internal carotid artery as demonstrated by the red arrow Red arrow demonstrating 70% stenosis of the left internal carotid artery. CT: computed tomography

**Figure 2:**
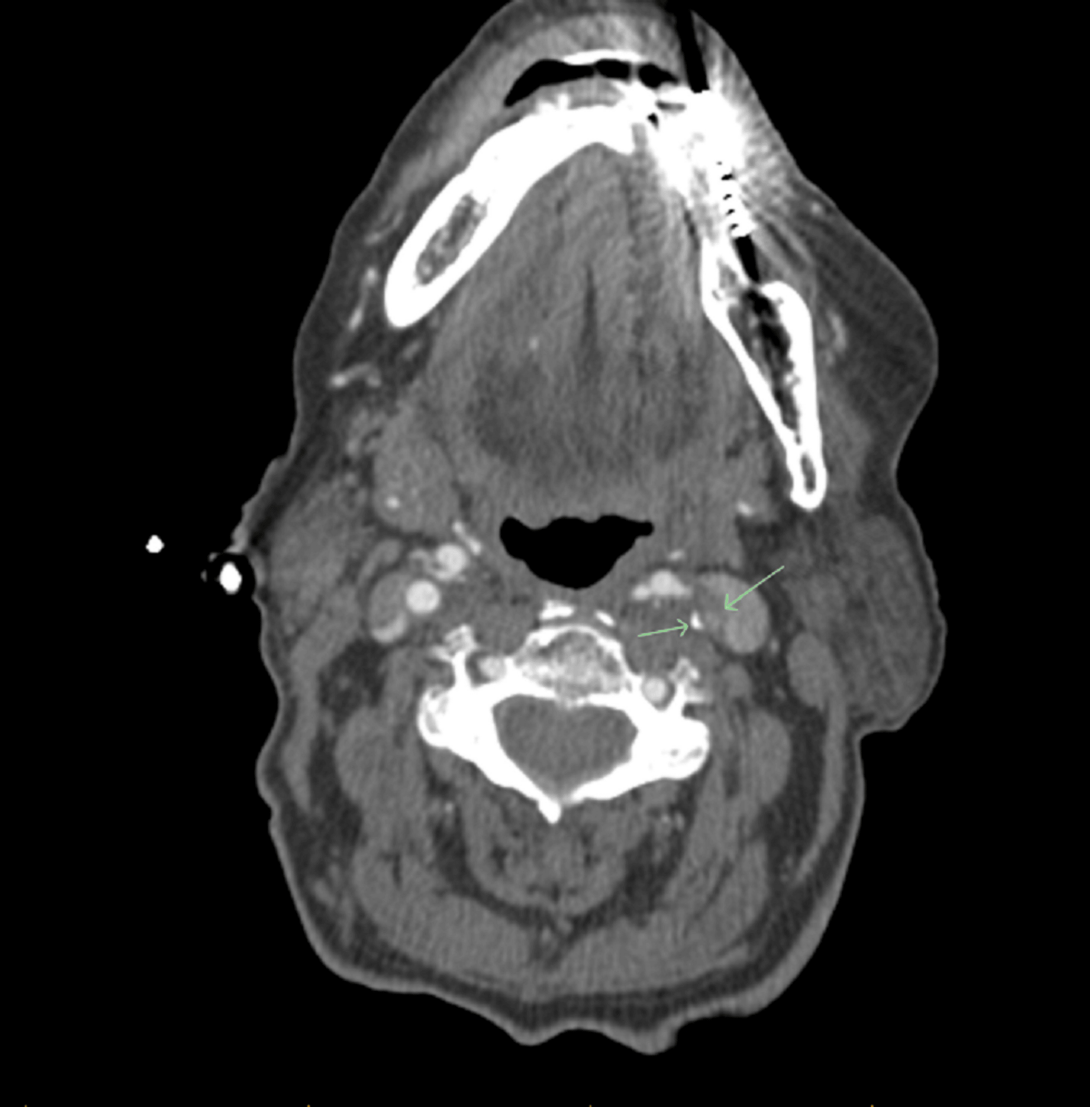
Coronal section of the CT angiogram with green arrows pointing to the left carotid artery stenosis Green arrows demonstrating 70% stenosis of the left internal carotid artery. CT: computed tomography

She had a multitude of tests done after arrival at the emergency room. Magnetic resonance imaging (MRI) of the brain showed chronic atrophic changes with no acute findings. A computed tomography (CT) angiogram of her head and neck was done, which revealed 70% stenosis of the left internal carotid artery (Figures 1, 2). She had no previous history of seizures and did have a complete recollection of events. Diagnosis of seizures was ruled out, and the episode was thought to be a TIA in view of underlying internal carotid artery stenosis. 

Since her neurologic deficit resolved, she refused hospitalization and opted to go home with outpatient physical therapy. She was started on clopidogrel. She was already on a low-dose aspirin (81 mg) and a high-intensity statin, which were continued. Table 1 shows detailed laboratory values obtained during her assessment in the emergency room. 

**Table 1:** Laboratory values obtained in the emergency room

Parameters	Laboratory values	Reference range
White blood cell (WBC) (/uL)	12,000	3,800-10,800
Hemoglobin (Hb) (g/dL)	12.8	11.1-15.1
Hematocrit (Hct) (%)	39.90	33-46
Platelet (/mL)	270,000	285,000
Sodium (meq/L)	137	137-145
Potassium (meq/L)	3.8	3.5-5.3
Chloride (meq/L)	101	98-107
Bicarbonate (meq/L)	23	21-32
Blood urea nitrogen (BUN) (mg/dL)	42	7-18
Creatinine (mg/dL)	5.3	0.52-1.04
Glucose (mg/dL)	239	74-106
Calcium (mg/dL)	8.5	8.4-10.2

After her initial episode, she frequently kept having similar episodes that coincided with dialysis treatments. She was sent to the emergency room on multiple occasions, and on each occasion, brain imaging failed to reveal any acute changes, and she chose to go home from the emergency room. Neurology also evaluated her in their outpatient clinic and advised her to continue aspirin, clopidogrel, and high-intensity statins with no further recommendations. After a couple of months of such episodes, she chose to stop dialysis treatments and remained off dialysis for about eight weeks.

During this time, her overall condition deteriorated, she became increasingly weak, started having episodic confusion, and became uremic. Her blood pressure became uncontrolled, and she had to be hospitalized for a hypertensive emergency. During hospitalization, she opted to restart hemodialysis, but this time for a much shorter duration. 

Cerebral shunting of blood flow while on dialysis was thought to be the possible reason for these episodes to coincide with hemodialysis. Modality change to peritoneal dialysis was discussed with the patient. The mechanism of peritoneal dialysis was explained, which theoretically has no risk of increased cerebral blood flow shunting, and she was much more likely to tolerate it. Given their age, she and her husband refused a change of modality to peritoneal dialysis.

**Figure 3:**
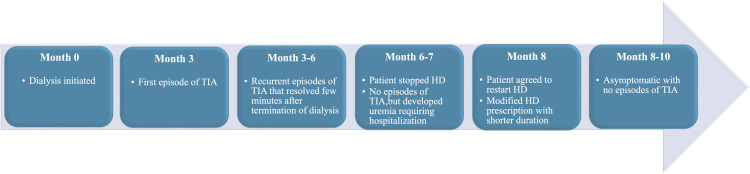
Diagram showing detailed chronology of events starting from initiation of dialysis to present TIA: transient ischemic attacks; HD: hemodialysis

The patient's dialysis prescription was modified. The duration of dialysis was changed to two hours as opposed to the standard four-hour treatment. She still gets her dialysis treatment three times a week. Blood flow rate was modified to 300 mL/min (as opposed to the standard 400 mL/min), and ultrafiltration rate was limited to less than 13 mL/kg/hr. Dialysate temperature was set at 37 degrees Celsius. She usually gains only 1 to 2 kg of weight between her dialysis treatments and has been able to maintain her volume status well. Her most recent dialysis adequacy was borderline at Kt/V 1.17 (goal Kt/V >1.2), and we have planned to increase the size of her dialyzer to help her meet the adequacy of dialysis treatment. She is tolerating this modified hemodialysis treatment well with no more episodes of TIA. A detailed series of these events is described in Figure 3. Table 2 describes current laboratory findings of patients with modified dialysis prescriptions. 

**Table 2:** Current laboratory findings with modified dialysis prescription

	Laboratory Values	Reference range
White blood cell (WBC) (in 1000/uL)	8.69	10.6
Hemoglobin (Hb) (g/dL)	8.6	11.1-15.1
Hematocrit (Hct) (%)	28.7	33-36
Platelet (1000/mL)	231	145-440
Sodium (meq/L)	138	137-145
Potassium (meq/L)	4.1	3.5-5.3
Chloride (meq/L)	101	98-107
Bicarbonate (meq/L)	24	21-32
Pre-dialysis blood urea nitrogen (BUN) (mg/dL)	48	6=19
Post-dialyisis BUN (mg/dL)	17	
Creatinine (mg/dL)	5.1	0.52-1.04
Glucose (mg/dL)	144	74-106
Albumin (g/dL)	3.6	3.2-5.2
Calcium (mg/dL)	8.5	8.4-10.2
Ferritin (ng/mL)	962	15-400
Iron (ug/dL)	30	30-160
Iron percent saturation (%)	13.4	15-50
Total iron-binding capacity (TIBC) (ug/dL)	223.1	250-450
Hemodialysis (HEMO) adequacy (Kt/V)	1.17	>1.2

## Discussion

Cerebrovascular disease ranks as the fifth leading cause of death in the United States [1]. The incidence of stroke is higher in patients with ESRD compared to the general population. A large observational cohort study conducted in Australia and New Zealand [2] using data obtained from their dialysis and transplant registry, hospital, and death records revealed a 2.5-to-11-fold increased risk of hospitalization with stroke in ESRD patients compared to the general population. The risk was higher in women compared to men and highest in young ESRD patients between 35 and 39 years of age [2]. Another prospective observational study conducted by Han et al. in Korea demonstrated a much higher risk in ESRD patients undergoing hemodialysis compared to peritoneal dialysis [3]. Our patient’s age, renal failure, need for hemodialysis treatment, female sex, and comorbidities like hypertension and diabetes were some of the factors that increased her risk of having acute cerebrovascular events. Her initial non-contrast CT scan and MRI of the brain were negative for acute hemorrhagic or ischemic events. Only after obtaining a neck CT angiogram did we notice her carotid stenosis as a possible factor causing her to have these recurrent episodes. This emphasizes the importance of thorough evaluation, especially when patients with high risk present with such episodes.

CVAs remain a major public health burden with very high morbidity. According to the latest Centers for Disease Control and Prevention (CDC) report published in 2022, every 40 seconds, someone in the United States has a stroke. It is a leading cause of long-term disability in the United States [4]. High blood pressure, high cholesterol, history of previous TIA or stroke, smoking history, diabetes, and obesity are some of the risk factors for stroke. A population-based cohort study conducted in the United States in 2003 suggested a five- to 10-fold higher risk of CVAs in hospitalized patients with ESRD on dialysis compared to the general population [5]. The risk was higher for both ischemic and hemorrhagic strokes; contrary to previous studies conducted in Japanese populations that revealed a higher risk of hemorrhagic stroke, the ischemic stroke risk was similar to that of the general population [6]. This may be because of the higher incidence of ESRD in the younger Japanese population with fewer comorbidities compared to that seen in the United States population.

There have been studies showing high calcified plaque burden in carotid and femoral arteries of patients with ESRD even without overt vascular disease occurrence [7]. This demonstrates a higher risk of progression of atherosclerotic disease amongst the ESRD population, which may be secondary to uremic conditions. Other dialysis-specific risk factors for CVA are frequent episodes of hemodynamic instability. Blood pressure fluctuations seen during dialysis treatment may compromise cerebral perfusion, thus increasing the risk of ischemic stroke. Anticoagulants used during dialysis treatment to keep blood from clotting in the circuit and increased bleeding risk due to uremia may also increase the risk of hemorrhagic stroke in patients on hemodialysis. Volume overload that happens between dialysis sessions can worsen cardiovascular disease and can, in turn, increase the risk of acute cerebral ischemic events. Chronic inflammation is a frequent occurrence in patients with ESRD [8] and is an independent risk factor for CVA [9].

Diagnosis is often delayed as similar symptoms often occur due to hemodynamic changes associated with dialysis treatments. Symptoms such as confusion, altered mental status, or dizziness can often be masked, especially in patients with uremia and associated cognitive impairment. Hypotension-related syncope or TIAs can go unnoticed or be misattributed to usual post-dialysis symptoms. Dialysis unit personnel are not equipped with the expertise required for thorough neurologic assessment, and this results in delayed referral to emergency medical services and delayed thrombolytic therapy, resulting in adverse patient outcomes. This case underscores the importance of recognizing alternative presentations, like acute cerebral events, that could be misrecognized as usual dialysis-related symptoms.

There is a lack of clear standardized guidelines for CVA treatment in ESRD patients needing dialysis. Thrombolytic therapy, as recommended in the general population for the treatment of ischemic stroke, can be challenging in patients on HD due to the high risk of hemorrhagic transformation, as they often need anticoagulants on dialysis. Physical rehabilitation is necessary after CVA becomes troublesome due to the need for frequent dialysis sessions and patient exhaustion seen post dialysis, limiting their ability to participate in therapy.

In our literature review, there has been only one reported case of similar episodes on dialysis. In that report, the patient’s symptoms resolved after changing the modality of dialysis to peritoneal [10]. Our patient, however, refused to change to peritoneal dialysis due to a lack of a support system, and so we decided to pursue the alternative solution of using shorter sessions with lower blood flow than the standard prescription.

As in our patient, carotid atherosclerosis plays a major role in the occurrence of CVA/TIA in patients on dialysis [7]. Secondary prevention strategies like tight blood pressure and blood glucose control, use of antiplatelet agents like aspirin and clopidogrel, and high-intensity statins are recommended, similar to the general population. Anticoagulation for atrial fibrillation warrants careful selection of agents like warfarin or apixaban (dose adjusted) with close monitoring of bleeding risk and dose adjustments based on serial laboratory values [11]. 

Carotid endarterectomy is a high-risk procedure for ESRD patients due to multiple comorbidities, and patients with ESRD are at a multifold higher risk of acute cardiac events. Although the perioperative stroke and death rate in patients with ESRD is similar to the general population, long-term survival after this procedure is poor, with a high four-year mortality rate in patients with ESRD [12]. Carotid artery stenting is a relatively low-risk procedure, but it is also shown to have an increased risk of post-procedural stroke and death [13]. Despite all the advances in current recommendations on CVA management, there remains much to be learned about treatment strategies for acute CVA and recurrent TIAs in patients with ESRD who remain at higher risk for these events. 

This rare case brings awareness of the need for tailored treatment strategies for acute cerebral events in the ESRD population to enhance patient outcomes.

## Conclusions

Treatment of acute cerebral events in dialysis patients lacks standardized guidelines and warrants an individualized approach. Modifying the dialysis prescription to the patient’s unique physiological and clinical context can significantly improve patient outcomes. This case underscores the importance of developing personalized treatment strategies in challenging situations where alternative modalities like peritoneal dialysis may not be an option. 
